# Female Mice Show Stronger Time‐of‐Day Modulation of Astrocytic Ca^2+^ Activity in the Sleep‐Regulatory Ventrolateral Preoptic Nucleus

**DOI:** 10.1002/glia.70181

**Published:** 2026-06-07

**Authors:** Félix Camille Bellier, Lou Zonca, David Holcman, Frédéric Chauveau, Nathalie Rouach, Armelle Rancillac

**Affiliations:** ^1^ Neuroglial Interactions in Cerebral Physiology and Pathologies, Center for Interdisciplinary Research in Biology, Collège de France, CNRS, INSERM Université PSL, PSL‐Neuro Paris France; ^2^ IRBA (Institut de Recherche Biomédicale Des Armées) Brétigny‐sur‐Orge France; ^3^ Toulouse Neuroimaging Center, INSERM UMR1214 Toulouse France; ^4^ Group of Applied Mathematics and Computational Biology, Ecole Normale Supérieure, PSL University Paris France

**Keywords:** AstroNet, calcium imaging, network, NREM sleep, sex differences, sleep regulation, VLPO

## Abstract

Astrocytes actively contribute to sleep regulation through intracellular calcium (Ca^2+^) signaling. Yet, whether astrocytic dynamics within sleep‐promoting hypothalamic nuclei vary across the nycthemeral cycle in a sex‐dependent manner remains unknown. The ventrolateral preoptic area (VLPO) is a key sleep‐promoting nucleus whose neuronal circuitry has been extensively characterized. However, the local astrocytic Ca^2+^ activity remains poorly defined. Here, we investigated astrocytic Ca^2+^ signaling in the VLPO of male and female mice across the nycthemeral cycle. Using two‐photon Ca^2+^ imaging in acute VLPO‐containing brain slices prepared at Zeitgeber Time (ZT)‐2, corresponding to the onset of the rest period, and ZT‐14, corresponding to the beginning of the active period, we combined single‐event analyses with graph‐based network approaches to characterize astrocytic activity across scales. At the level of individual events, spontaneous astrocytic Ca^2+^ dynamics exhibited marked state dependence and sexual dimorphism. In males, Ca^2+^ events were smaller and faster at ZT‐14 than at ZT‐2 (shorter duration and accelerated rise and decay time). In contrast, in females, ZT‐14 was characterized by increased event amplitude and frequency, consistent with upregulated Ca^2+^ signaling during the active phase. At the network level, functional connectivity remained stable in males. Conversely, females exhibited robust network remodeling at ZT‐14, including increased astrocyte recruitment, higher node degree of correlations, and a marked rise in the number and proportion of highly connected astrocytes. Together, these findings reveal sex‐specific astrocytic signaling strategies in the VLPO across the nycthemeral cycle and underscore the need to incorporate sex as a biological variable in astrocyte‐based sleep research.

## Graphical abstract

- VLPO astrocytes exhibit sex‐specific Ca^2+^ dynamics across the nycthemeral cycle.
- Males show faster Ca^2+^ kinetics, while females display increased amplitude, frequency, and network coupling at Zeitgeber Time (ZT)‐14.
- Sex is a key variable in astrocyte‐based sleep regulation mechanisms.

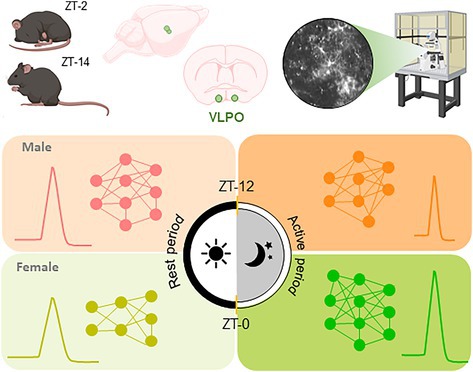

## 1 Introduction

Astrocytes are increasingly recognized as active regulators of brain state, exerting dynamic control over synaptic transmission, metabolic coupling, and neuromodulatory signaling. Central to these functions is the intracellular calcium (Ca^2+^) signaling, which enables astrocytes to integrate local inputs and engage downstream effector mechanisms, including gliotransmitter release (Araque et al. 2014). A growing body of evidence indicates that astrocytic Ca^2+^ dynamics are tightly linked to sleep homeostasis and vary across vigilance states, often preceding neuronal state transitions and exhibiting patterns distinct from those of neuronal activity (Poskanzer and Yuste 2016; Bojarskaite et al. 2020; Ingiosi et al. 2020).

Astrocytic Ca^2+^ signaling during sleep is highly region‐specific. Cortical and hippocampal astrocytes show pronounced reductions in Ca^2+^ activity during non‐rapid eye movement (NREM) sleep, whereas astrocytes in subcortical regions such as the hypothalamus and brainstem display distinct and sometimes more stable Ca^2+^ dynamics, suggesting circuit‐dependent astrocytic operating modes (Tsunematsu et al. 2021; Vaidyanathan et al. 2021). However, astrocytic Ca^2+^ activity within defined sleep‐regulatory hypothalamic nuclei remains poorly characterized.

The ventrolateral preoptic area (VLPO) is a key sleep‐promoting hypothalamic nucleus whose neuronal circuitry has been extensively studied, yet the contribution of local astrocytes is only beginning to emerge. Recent work has shown that VLPO astrocytes can actively promote sleep through ATP‐dependent mechanisms, implicating astrocytic Ca^2+^ signaling as a regulator of VLPO output and sleep pressure (Scharbarg et al. 2016, 2023; Bellier et al. 2025). Whether astrocytic Ca^2+^ dynamics in the VLPO vary across the nycthemeral cycle and how they are organized at both the single‐cell and network levels remains unknown.

An additional and largely overlooked dimension in astrocyte‐based sleep research is biological sex. Sleep architecture and sleep homeostasis differ between males and females in rodents and humans, yet most studies of astrocytic Ca^2+^ signaling during sleep have relied on male‐only or non‐stratified cohorts (Ingiosi and Frank 2023). As a result, potential sex‐specific astrocytic signaling strategies may have been overlooked.

Here, we aim to investigate astrocytic Ca^2+^ dynamics within the VLPO of male and female mice across the nycthemeral cycle. We used two‐photon Ca^2+^ imaging in acute VLPO‐containing brain slices prepared at two opposing circadian time points in healthy adult mice. We combined single‐event analyses with graph‐based network approaches to characterize astrocytic signaling across scales and hypothesize that astrocyte Ca^2+^ dynamics may differ according to sex and circadian time of day, potentially underpinning behavioral sex differences in sleep regulation.

## 2 Methods

### 2.1 Animals

The GFAP‐CreERT2 line was crossed with the Cre‐dependent Ai95(RCL‐GCaMP6f)‐D reporter line to generate GFAP‐CreERT2::GCaMP6f mice and express the Ca^2+^ indicator in astrocytes (Madisen et al. 2015). The Aldh1l1‐EGFP reporter mice correspond to the *Tg(Aldh1l1‐EGFP/Rpl10a)JD133Htz* line (Doyle et al. 2008), and the Aldh1l1‐CreERT2 mice correspond to the *Tg(Aldh1l1‐Cre/ERT2)02Kan* line (Srinivasan et al. 2016).

The GFAP‐CreERT2::GCaMP6f mice were used for biphotonic Ca^2+^ imaging. In this mouse line, the Cre recombinase (Cre), fused to a mutant form of the estrogen receptor ERT2, requires tamoxifen to be active in GFAP‐positive cells only. To induce GCaMP6f expression, GFAP‐CreERT2::GCaMP6f mice were injected intraperitoneally with tamoxifen (100 mg/kg/day for 3 days). All mouse lines were maintained on a C57BL/6J genetic background and used in both sexes between P50 and P60. Animals were sampled at ZT‐2 and ZT‐14 (males: *n* = 7 at ZT‐2 and *n* = 7 at ZT‐14; females: *n* = 7 at ZT‐2 and *n* = 6 at ZT‐14), yielding 67, 70, 65, and 61 events, respectively.

Mice were housed in temperature‐controlled (20°C–22°C) and light‐tight ventilated cabinets, under a 12 h light–dark cycle with *ad libitum* access to food and water. The beginning of the day (lights‐on, rest phase) was at 08:00 a.m. (Zeitgeber time 0; ZT‐0). The beginning of the night (lights‐off, active phase) was at 08:00 p.m. (ZT‐12).

All animals were removed from the animal facility at either ZT‐2 or ZT‐12 and brought to the laboratory 10 min later. They were then rapidly sacrificed following procedures that were conducted in strict compliance with our institutional protocols and approved by the European Community Council Directive of 1 January 2013 (2010/63/EU) and the local ethics committee (C2EA‐59, “Paris Centre et Sud”) and local guidelines for the ethical treatment of animal care (Center for Interdisciplinary Research in Biology in College de France, France). The number of animals used in our study was kept to a minimum.

### 2.2 Preparation of Acute Hypothalamic Slices

After decapitation, brains were quickly extracted and submerged in cold slicing artificial cerebrospinal fluid (aCSF, 4°C) for 2–3 min before sectioning, containing (in mM): 130 NaCl; 5 KCl; 2.4 CaCl_2_; 20 NaHCO_3_; 1.25 KH_2_PO_4_; 1.3 MgSO_4_; 10 d‐glucose; 15 sucrose; and 1 kynurenic acid (pH = 7.35). Brains were constantly oxygenated with 95% O_2_–5% CO_2_. Coronal brain slices (300 μm thick) containing the VLPO were cut with a vibratome (VT2000S; Leica) and transferred to a constantly oxygenated (95% O_2_–5% CO_2_) holding chamber containing aCSF for at least 1 h for recovery.

### 2.3 Two‐Photon Ca^2+^ Imaging

Individual brain slices were transferred to a submerged recording chamber maintained at 32°C and continuously perfused with oxygenated aCSF. Slices were allowed to equilibrate for at least 10 min before imaging to ensure adaptation to the recording temperature. Imaging was performed using an upright two‐photon microscope (Axio Examiner Z1, Carl Zeiss) equipped with a 2‐photon illumination system (3i Intelligent Imaging) and a Chameleon Ultra II Ti:sapphire laser (Coherent). GCaMP6f‐expressing astrocytes were excited at 920 nm, and fluorescence was collected through a 525/40 nm emission filter and a 580 nm dichroic mirror. Images were acquired at 3 Hz using a 40× water‐immersion objective (NA 0.95, Olympus). Image sequences were pre‐processed offline with Slidebook imaging software (3i), including the application of a median filter to improve image quality. One ROI per slice was imaged for 5 min. No more than four slices were used per experimental day, and no difference was observed between the first and last recordings of the day.

### 2.4 Astrocytic Ca^2+^ Analysis and Functional Network Reconstruction

We quantify astrocytic Ca^2+^ transients from fluorescent time‐series data using our previously developed Matlab‐based algorithm, AstroNet (Zonca et al. 2025) (https://github.com/louzonca/AstroNet). Briefly, the algorithm processes the Ca^2+^ fluorescent signals summed and normalized for each ROI over a 5 min recording period. Signals were averaged over 30 s intervals to detect the position of active astrocytes. Once each contributing individual astrocyte was identified, Ca^2+^ transients were extracted following baseline correction to remove slow fluorescence fluctuations, enabling a spatio‐temporal segmentation of the activity. This segmentation serves two purposes.

First, it allows quantification of individual Ca^2+^ event parameters, including event duration (temporal extent of each Ca^2+^ transient), amplitude (peak fluorescence relative to the baseline, ΔF/F), event rise time (time from the baseline to the first peak maximum), decay time (time from the peak toward baseline), and event frequency (number of Ca^2+^ events detected during the recording period).

Second, the detected astrocytes and their activity relationships were used to reconstruct a functional network in which each node represents an astrocyte and edges correspond to correlations between astrocytic Ca^2+^ signals. This graph‐based representation allows characterization of astrocytic network organization. Highly connected cells were defined as hub astrocytes, corresponding to nodes functionally associated with at least 60% of the other astrocytes in the network.

We further quantified several network metrics. Path length was defined as the shortest distance between pairs of correlated astrocytes within the reconstructed network. The mean node degree was calculated as the average number of correlations per astrocyte. Finally, we quantified both the number and the proportion of highly correlated astrocytes within each functional network.

### 2.5 Statistics

All dot plots represent single events or means per astrocyte or ROI when specified. No statistical differences between slices or animals were found. Bar graphs (right panels) represent the mean elements shown on the left panels ± standard error of the mean (SEM). Statistical significance was assessed using appropriate tests based on data distribution and experimental design. As data distributions did not meet the assumptions of normality and homogeneity of variance using Shapiro–Wilk and Brown–Forsythe tests, group comparisons were conducted using a non‐parametric Kruskal–Wallis test, followed by Dunn's multiple comparisons test. Statistical comparison of delta measurements was performed using Welch's *t*‐tests, computed analytically from group means, standard deviations, and sample sizes. All statistical analyses were conducted using GraphPad Prism (version 8.0.2) and Excel. The specific tests applied are indicated in the corresponding figure legends. Significance was set at *p* < 0.05 and expressed as follows: *p < 0.05, **p < 0.01, ***p < 0.001.

Outliers were detected with the median‐absolute‐deviation (MAD) criterion: the sample median m was first estimated, the absolute residuals ∣xi−m∣ were then computed for each observation, and their median yielded the MAD, a robust scale estimator (Leys et al. 2013). Any observation lying outside the interval *m* ± 3.75 MAD was classified as an outlier and excluded from subsequent analyses.

## 3 Results

Sleep exhibits sex‐specific features, likely reflecting distinct underlying regulatory mechanisms. Astrocytes have recently emerged as active contributors to sleep regulation, yet whether their activity differs between sexes remains unknown. We therefore hypothesized that astrocytic Ca^2+^ dynamics within the ventrolateral preoptic area (VLPO), a key sleep‐promoting nucleus, might vary across the light–dark cycle in a sex‐dependent manner. To test this, we performed two‐photon Ca^2+^ imaging in acute VLPO‐containing brain slices prepared around ZT‐2, falling within the light phase (elevated sleep propensity), and ZT‐14, falling within the dark phase (elevated wake propensity), under standard light–dark conditions.

**FIGURE 1:**
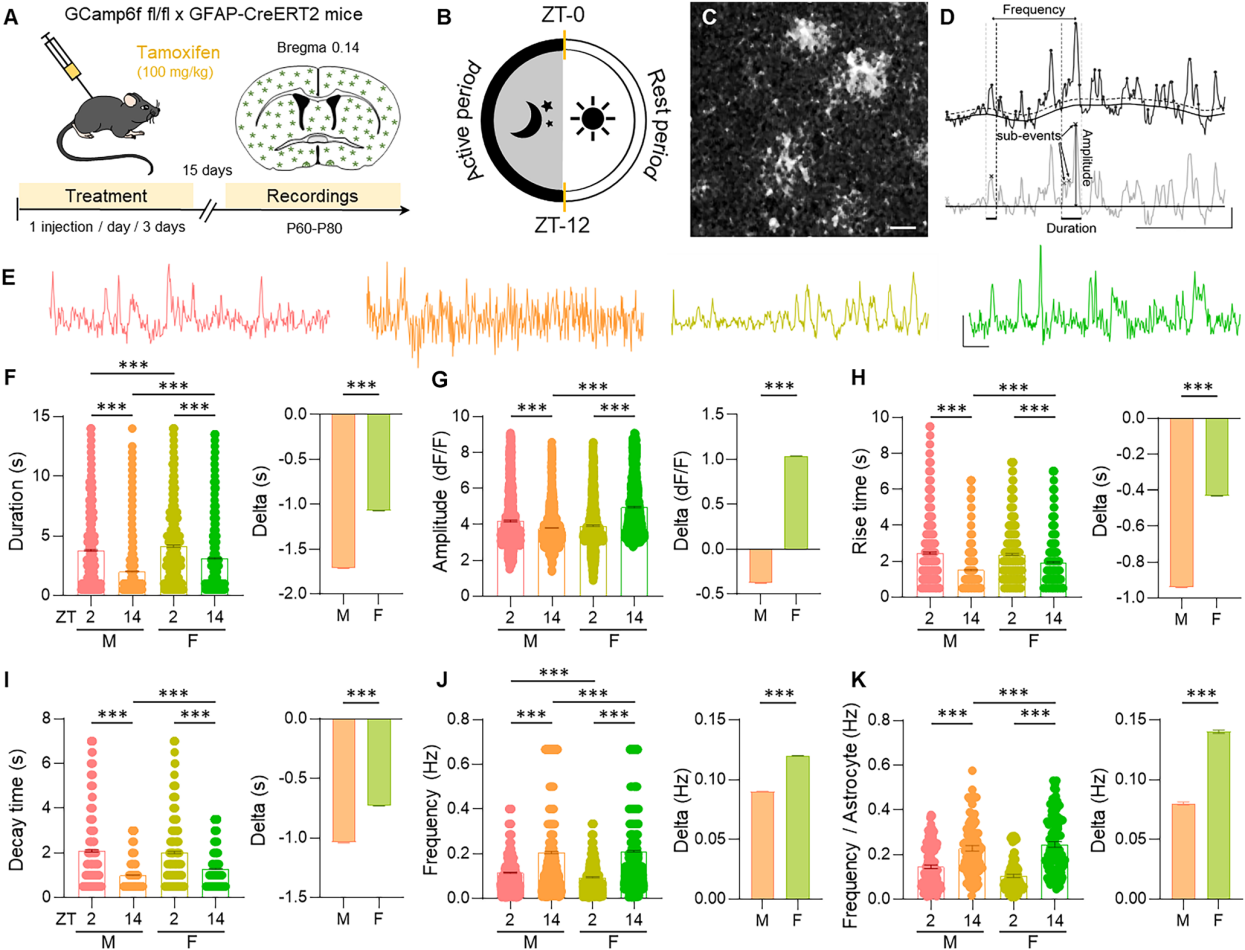
Single‐event analysis of Ca^2+^ signals in VLPO astrocytes from male and female mice at ZT‐2 and ZT‐14. (A) Experimental design. (B) Schematic illustration of the zeitgeber time (ZT). (C) Representative field of view for two‐photon Ca^2+^ imaging. Scale bar: 20 μm. (D) Selected steps of the analysis workflow using the AstroNet algorithm, including baseline correction, peak detection, and the determination of event amplitude, frequency, and duration. Scale bars: 3 dF/F and 50 s. (E) Representative spontaneous Ca^2+^ traces from individual astrocytes in male and female mice at ZT‐2 and ZT‐14, illustrating the sex‐ and ZT‐dependent differences. Scale bars: 5 dF/F and 20 s. (F–K) Individual data points per ROI are represented by colored circles, and bars indicate the mean ± sem values for both sexes across ZT: For event duration (F), amplitude (G), rise time (H), decay time (I), event frequency (J), and per‐astrocyte event frequency (K). Males: *N* = 7 at ZT‐2 and at ZT‐14; females: *N* = 7 at ZT‐2 and *n* = 6 mice at ZT‐14; yielding 67, 70, 65, and 61 events, respectively. ***p < 0.001 with Kruskal‐Wallis tests, followed by Dunn's multiple comparisons test, and Welch's t‐tests.

We identified numerous differences in astrocytic Ca^2+^ activity, both between the sexes and across the nycthemeral cycle, by focusing on individual spontaneous Ca^2+^ events and network functional organization. First, we examined individual Ca^2+^ events, using our previous AstroNet algorithm (Figure 1) (Zonca et al. 2025). We quantified Ca^2+^ event duration, a key descriptor of astrocytic signaling dynamics, since longer‐lasting Ca^2+^ elevations may engage broader downstream pathways and could contribute to the modulation of local sleep–wake circuitry (Ingiosi et al. 2020; Bellier et al. 2025).

Representative two‐photon Ca^2+^ imaging recordings are provided as Supporting Information (Videos [Link], [Link]) for male and female mice at ZT‐2 and ZT‐14 respectively.

In the VLPO, we observed longer spontaneous events at ZT‐2 than at ZT‐14. In males, the mean event duration drops by ≈45%, whereas in females it decreases only by ≈26% (Table 1, Figure 1F). This indicates stronger nycthemeral modulation in males. Moreover, females maintain longer events at both time points.

**TABLE 1:** Astrocytic event parameters.

Parameter	ZT‐2 Male	ZT14 male	ZT‐2 female	ZT14 female
Duration (s)	3.80 ± 0.07	2.01 ± 0.04	4.16 ± 0.09	3.12 ± 0.05
Amplitude (dF/F)	4.19 ± 0.05	3.81 ± 0.02	3.93 ± 0.05	4.97 ± 0.04
Rise time (s)	2.46 ± 0.05	1.52 ± 0.03	2.38 ± 0.06	1.95 ± 0.03
Decay time (s)	2.09 ± 0.04	1.01 ± 0.02	2.02 ± 0.05	1.28 ± 0.02
Frequency (Hz)	0.12 ± 0.00	0.21 ± 0.00	0.09 ± 0.00	0.21 ± 0.00
Frequency/astrocyte (Hz)	0.15 ± 0.01	0.23 ± 0.01	0.10 ± 0.01	0.25 ± 0.01

*Note:* Mean ± SEM.

We next quantify the Ca^2+^ event amplitude, reflecting the intensity of astrocytic recruitment and the potential downstream signaling effects (Table 1, Figure 1G). In males, amplitude showed a modest attenuation of ~6% across the nyctemeral cycle, whereas females exhibit a marked rise of ~21%. Thus, across the nycthemeral cycle, males primarily display a reduction in event amplitude across time of the day, while females shift toward higher‐amplitude events, suggesting opposite regulatory mechanisms.

We then extracted rise times, defined as the interval between the Ca^2+^ baseline and the first maximum peak. Shorter rise times suggest faster recruitment of astrocytic signaling. In males, the rise time is shortened by ~38%, which is again double that of the shortened ~18% observed in females, despite a similar rise time at ZT‐2 (Figure 1H). Similarly, we quantified the decay time of Ca^2+^ and found comparable values between males and females at ZT, such as kinetic reconfiguration. However, the decrease at ZT‐14 is still significantly higher in males than in females (Figure 1I)s, suggesting that time‐of‐day‐dependent reconfiguration of astrocyte Ca^2+^ event kinetics differs between sexes in the VLPO. Finally, to complete our description of single astrocytic events, we quantified the event frequency. We first measured frequency (Figure 1J). In males, the frequency increased by ~75% after the rest period. In females, the frequency increased by ~140%. We then measured frequency per astrocyte, which shows a marked difference. In males, the rate rose by ~50%, whereas in females, it increased by ~140%. Thus, both sexes showed higher event propensity at ZT‐14, with a much larger increase in females. The final per‐astrocyte frequency was also slightly higher in females (0.24 vs. 0.23; Figure 1K). Thus, both sexes show a nycthemeral upshift in event propensity at ZT‐14, with a larger relative gain in females and a slightly higher final per‐astrocyte frequency.

Taken together, VLPO astrocytes exhibit marked state dependence and clear sex differences regarding single astrocyte events: Males show stronger nycthemeral modulation of duration and onset, while females display higher amplitudes at ZT‐14 and larger relative increases in frequency. These patterns indicate sex‐dependent tuning of astrocytic signaling across vigilance states.

**FIGURE 2:**
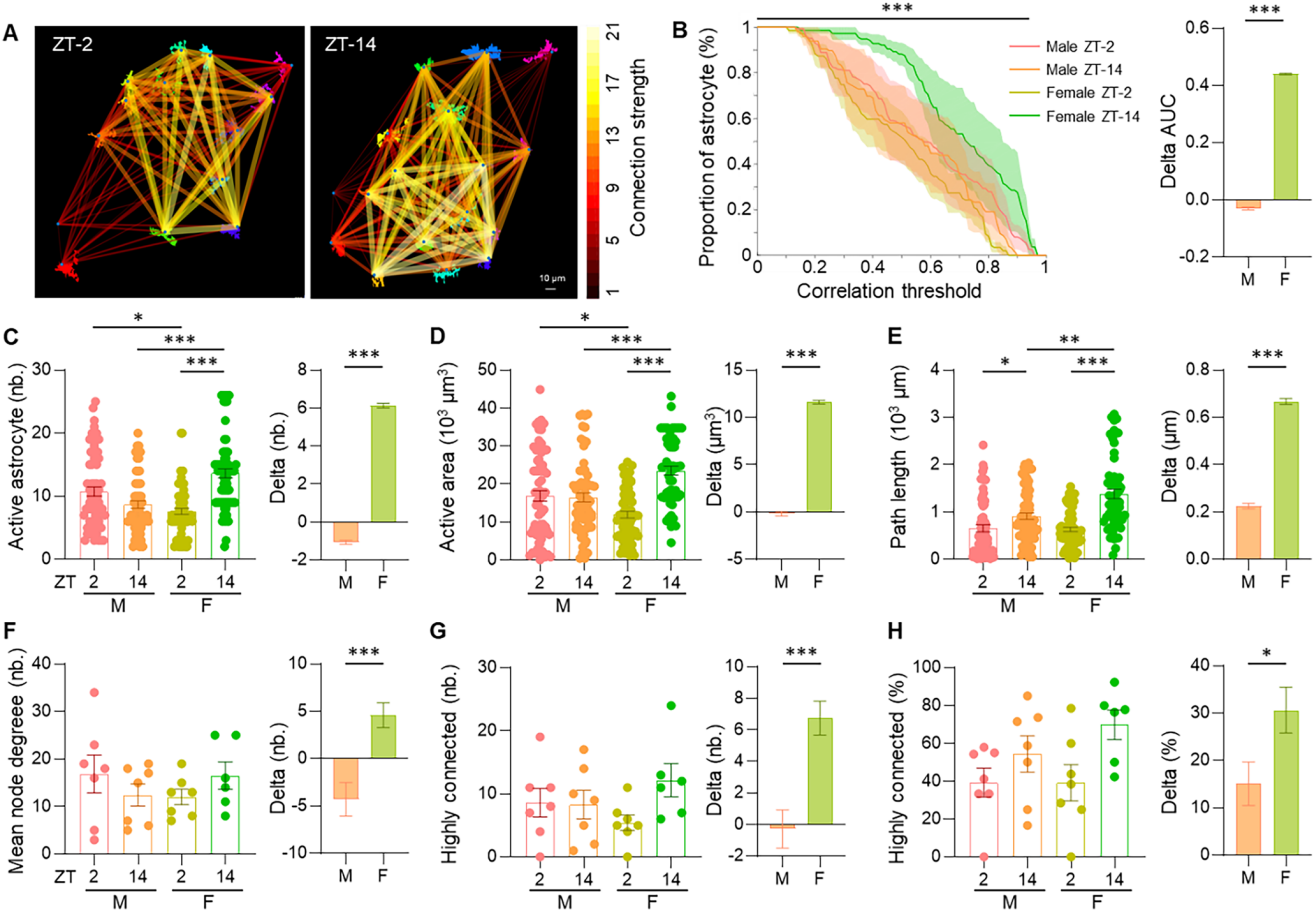
AstroNet‐based network analysis of VLPO astrocytic Ca^2+^ activity in male and female mice at ZT‐2 and ZT‐14. (A) Representative reconstructed functional graphs, with edges color‐coded by connectivity strength (yellow, stronger; red, weaker). (B) Left panel: The correlation curves. A steeper decay indicates a less robust connected network. Right panel: Difference in the area under the curve between ZT‐2 and ZT‐14 (ΔAUC) for males and females. (C‐H) Summary plots and means showing sex‐dependent differences across ZT, and their differences across ZT for: The number of active astrocytes per ROI (C), their corresponding active area (D), individual path lengths per ROI (E), mean node degrees (F), number of highly connected astrocytes per ROI (G), proportion of highly connected astrocytes per ROI (> 60%) (H). Males: *N* = 7 at ZT‐2 and at ZT‐14; females: *N* = 7 at ZT‐2 and *n* = 6 mice at ZT‐14. *p < 0.05, ** < 0.01, and *** < 0.001 with Kruskal‐Wallis tests, followed by Dunn's multiple comparisons test, and Welch's *t*‐tests.

To further characterize astrocytic activity at the network level, we performed a pairwise Pearson correlation analysis of astrocytic Ca^2+^ signals using the AstroNet algorithm (Figure 2). To quantify the overall level of functional coupling between active astrocytes, we computed the correlation curve for each experimental condition (Figure 2B) and used the area under the curve (AUC) as an integrative measure of global network functionality. This curve reveals the persistence of the network across increasing detection threshold: in a highly persistent astrocytic ensemble, connectivity remains elevated even under more stringent correlation criteria. Thus, the AUC provides a measure of the stability and robustness of the functional astrocytic network: In males, AUC values were comparable at ZT‐2 (0.58 ± 0.26) and ZT‐14 (0.55 ± 0.26), suggesting that local astrocytic network functional connectivity remains largely stable across the nycthemeral cycle. In contrast, female mice exhibited a robust modulation, with AUC increasing from 0.50 ± 0.06 at ZT‐2 to 0.71 ± 0.08 at ZT‐14, corresponding to a ~42% increase. This indicates increased functional coupling between astrocytes in the VLPO, particularly at the beginning of the wake period as the AUC at ZT‐2 is similar to that of males.

In males, we found that network parameters were largely stable across the nycthemeral cycle, with no or only small changes between ZT‐2 and ZT‐14 in the number of active astrocytes, their active area, path length, mean node degree, and the number and proportion of highly connected astrocytes (Table 2, Figure 2 C—H). Consistently, analysis of the correlation curves revealed no significant difference between ZT‐2 and ZT‐14, indicating relatively stable functional correlations between astrocytes in males.

**TABLE 2:** Astrocytic functional network organization.

Parameter	ZT‐2 male	ZT14 male	ZT‐2 female	ZT14 female
Active astrocyte (nb)	10.72 ± 0.72	8.68 ± 0.61	7.62 ± 0.48	13.65 ± 0.73
Active area (10^3^ μm^3^)	16.85 ± 1.39	16.73 ± 1.17	11.71 ± 0.88	23.24 ± 1.18
Path length (10^3^ μm)	0.65 ± 0.08	0.91 ± 0.07	0.63 ± 0.05	1.38 ± 0.10
Mean node degree (nb)	16.86 ± 4.00	12.43 ± 2.33	12.00 ± 1.62	16.50 ± 2.91
Highly connected (nb)	8.57 ± 2.28	8.29 ± 2.26	5.43 ± 1.25	12.17 ± 2.63
Highly connected (%)	39.29 ± 7.62	54.37 ± 9.67	39.19 ± 9.58	69.81 ± 7.89

*Note:* Mean ± SEM.

In contrast, female mice displayed marked nycthemeral changes in astrocytic network properties between ZT‐2 and ZT‐14, consistent with a substantial strengthening and reshaping of functional connectivity during the wake period. This change was accompanied by a stronger functional connectivity and a sharp rise in the number of active astrocytes by ~80% and in the active astrocytic area by ~100%. The network topology was also profoundly modified, with a strong increase in path length by ~110% and a concomitant increase in mean node degree by ~40%, indicating denser local connectivity alongside a reorganization of long‐range integration. In parallel, the number of highly connected astrocytes more than doubled by ~125%, and their proportion increased substantially by ~80% (Table 2, Figure 2 C—H).

Collectively, these findings reveal a robust, sex‐specific modulation of VLPO astrocytic network organization across the nycthemeral cycle, with large and selective strengthening of functional connectivity in females at ZT‐14, while males maintain a comparatively stable network architecture.

## 4 Discussion

Growing evidence indicates that astroglial Ca^2+^ signaling contributes directly to sleep homeostasis rather than simply reflecting local neuronal activity (Ingiosi et al. 2020; Kim et al. 2020). Whereas previous studies mainly focused on mixed or single‐sex cohorts, our study reveals a marked sexual dimorphism in spontaneous VLPO astrocytic Ca^2+^ dynamics across the nycthemeral cycle. Although both sexes exhibited ZT‐dependent modulation of single‐event features between ZT‐2 and ZT‐14, the magnitude and pattern of these changes differed substantially between sexes.

In males, changes were primarily expressed through alterations in event kinetics, characterized by shorter event duration and faster rise and decay times. In contrast, females exhibited a stronger recruitment of high‐amplitude, high‐frequency events, along with a large‐scale reorganization of the functional astrocytic network.

### 4.1 Sex‐Dependent Tuning of Single‐Event Astrocytic Signaling

Here, ZT‐dependent changes observed in the VLPO suggest that astrocytes undergo state‐related remodeling of their spontaneous Ca^2+^ activity. Notably, we found that the reduction in astrocytic Ca^2+^ event duration between ZT‐2 and ZT‐14 is substantially larger in males than in females, indicating a stronger nycthemeral modulation of astrocytic event kinetics in males. This shortening is associated with faster rise and decay times, indicating a more rapid recruitment and termination of astrocytic Ca^2+^ signals at ZT‐14. Such kinetic shifts suggest a change from a prolonged, integrative signaling mode to a more temporally constrained mode during activity, depending on the time of day.

The duration of astrocytic Ca^2+^ events is a critical determinant of gliotransmitter release, as sustained Ca^2+^ elevations are required to engage Ca^2+^‐dependent release mechanisms and to promote spatial propagation of Ca^2+^ signals toward functional release sites. Accordingly, such kinetic reconfiguration may be relevant for local regulation of VLPO output, as astrocytic Ca^2+^ signaling can trigger gliotransmission and metabolic support over timescales compatible with sleep pressure dynamics, notably through ATP and adenosine‐related mechanisms that modulate neuronal excitability and sleep homeostasis (Scharbarg et al. 2016, 2023; Ingiosi and Frank 2023; Bellier et al. 2025).

Changes in event amplitude and frequency further support the existence of sex‐specific mechanisms by which astrocytes encode and convey vigilance state. In males at ZT‐14, event amplitude decreases slightly while event frequency increases, suggesting that wake‐related signaling is conveyed predominantly by more frequent activation. In contrast, females exhibit both an increase in event amplitude and frequency at ZT‐14, suggesting a coordinated recruitment of more intense astrocytic activity during the wake period. These findings are consistent with previous studies showing that astrocytic Ca^2+^ event frequency and amplitude vary across sleep and wake states (Tsunematsu et al. 2021; Ingiosi and Frank 2023).

Previous work by Tsunematsu et al. (2021) indeed reported that astrocytic Ca^2+^ levels decrease markedly in the cortex and hippocampus during NREM sleep, while remaining relatively stable in the hypothalamus and brainstem. During REM sleep, astrocytic Ca^2+^ levels were consistently decreased across brain regions. However, these conclusions were drawn exclusively from male animals. By explicitly including both sexes, our study reveals that the apparent stability of hypothalamic astrocytic Ca^2+^ activity reported previously was likely driven by male‐specific patterns, thereby probably masking sex‐specific dynamics.

Together, these observations emphasize that astrocytic operating modes and their time‐of‐day‐dependent activity depend not only on brain regions but also on sex. This highlights the need for future studies to systematically integrate sex as a biological variable to fully capture the diversity of astrocyte signaling strategies underlying sleep regulation.

### 4.2 Female‐Specific Strengthening of Network Coupling at ZT‐14

Network‐level analyses sharpen this divergence. In males, the correlation‐curve AUC remained relatively stable from ZT‐2 to ZT‐14, indicating that global coupling between active astrocytes is preserved despite pronounced changes in event kinetics. These results suggest that nycthemeral modulation in males is more likely driven by intracellular or highly local mechanisms, such as shifts in receptor recruitment, Ca^2+^ buffering or clearance, or preferential engagement of subcellular microdomains rather than by a broader reorganization of inter‐astrocytic coupling or network‐level coordination.

In contrast, females showed a strong increase in AUC at ZT‐14, indicating enhanced synchrony or functional coupling among active astrocytes during the wake period. This was accompanied by a marked rise in the number of active astrocytes and active area, a higher mean node degree, and a pronounced increase in the number and proportion of highly connected astrocytes, consistent with the emergence of more hub‐like astrocytes and denser local functional correlation network. Together, these changes support the idea that, in females, astrocytic activity at ZT‐14 is not only increased in amplitude but also displays enhanced spatial coordination, reflecting a more network‐integrated level of organization.

### 4.3 Sex‐Specific Hormonal Modulation of Sleep

Sex‐specific patterns of sleep and astrocytic activity may arise from differential hormonal modulation of sleep–wake regulatory systems. Numerous publications on rodents indicate that baseline sleep architecture differs between males and females, and that both sleep homeostasis and arousal responses exhibit sexual dimorphism and sensitivity to gonadectomy and hormone replacement (Choi et al. 2021; Dib et al. 2021). In addition, astrocytes express receptors for gonadal hormones, positioning them as potential cellular substrates through which hormonal signals may influence sleep‐regulatory circuits.

In line with this view, female C57BL/6J mice exhibit reduced total sleep and NREM sleep, accompanied by increased wakefulness, a greater proportion of REM sleep, and shorter NREM bout lengths compared to males (Mannino et al. 2024). Notably, several of these sex differences persist after gonadectomy, suggesting that both hormonal and non‐hormonal mechanisms contribute to the sexual differentiation of sleep phenotypes (Ehlen et al. 2013; Choi et al. 2021).

Beyond gonadal hormones, noradrenergic signaling represents another mechanism through which time‐of‐day‐dependent differences in astrocytic Ca^2+^ dynamics could arise. Noradrenaline, released from locus coeruleus projections, is a potent activator of astrocytic Ca^2+^ signaling via α 1‐adrenoceptors, driving large‐scale coordinated Ca^2+^ responses across astrocytic populations in vivo (Ding et al. 2013; Wahis and Holt 2021; Lines et al. 2025) and this system plays a central role in sleep–wake regulation and arousal (Van Egroo et al. 2022; Antila et al. 2022). LC‐NE tonic firing rate is highest during wakefulness, lower during NREM sleep, and virtually silent during REM sleep, such that noradrenergic tone varies substantially across the nycthemeral cycle in a manner that broadly mirrors the ZT‐2/ZT‐14 contrast examined here. In the acute‐slice preparation used here, noradrenergic inputs are cut, and the spontaneous Ca^2+^ activity recorded therefore reflects intrinsic astrocytic properties independent of ongoing noradrenergic drive. Nevertheless, ZT‐dependent differences in noradrenergic tone in vivo may exert lasting effects on astrocytic gene expression, receptor availability, or intracellular signaling competence that effectively ‘prime’ astrocytes in a state‐dependent manner before slice preparation and potentially contribute to the ex vivo signatures reported here. This interpretation is consistent with the known capacity of prior LC‐NE activity to modulate astrocytic responsiveness on timescales extending beyond the immediate period of noradrenaline release (Ding et al. 2013; Wahis and Holt 2021).

An additional dimension concerns sex differences in the LC‐NE system itself. Emerging evidence indicates that LC activity, noradrenergic receptor expression, and sensitivity to stress and arousal differ between males and females, though the mechanistic basis of these differences remains incompletely characterized. Whether such sex‐dependent variation in LC‐NE output is reflected in differential priming of VLPO astrocytes and whether this contributes to the female‐specific strengthening of the network coupling observed at ZT‐14 in our study represents an open and particularly interesting question for future in vivo investigations combining LC activity monitoring with astrocytic Ca^2+^ imaging in both sexes.

Within this framework, the female‐specific increase in astrocytic network coupling observed at ZT‐14 may reflect a hormone‐dependent facilitation of astrocytic recruitment, inter‐astrocytic coordination, and/or gliotransmission within the VLPO. Although the slice preparation used here does not allow a direct association between astrocytic Ca^2+^ dynamics and concurrent sleep state, our results demonstrate that sex and ZT are significant determinants of astrocytic Ca^2+^ activity in this key sleep‐regulatory nucleus.

### 4.4 Translational Perspectives and Relevance to Human

Although cross‐species translation must be cautious (nocturnal mice vs. diurnal humans; distinct endocrine milieus; strong sociocultural modulation in humans), the concept of sex‐specific sleep regulatory mechanisms is shared across species. In humans, women report a greater need for sleep and, across objective measures, often exhibit longer total sleep time, better sleep efficiency, and more slow‐wave sleep than men. Paradoxically, women also report more sleep complaints and have higher insomnia prevalence (Baker et al. 2025; dos Silva et al. 2025).

### 4.5 Time of Day as an Experimental Variable in Studies

Beyond its implications for sleep regulation, the present study highlights a methodological consideration relevant to the broader astrocyte field. Our results demonstrate that astrocytic Ca^2+^ dynamics in the VLPO vary substantially with Zeitgeber time, and that these differences persist in the acute‐slice preparation. This raises the possibility that astrocyte imaging studies, slice physiology experiments, and downstream molecular analyses that do not control for the time of sacrifice or recording may be unintentionally confounded by time‐of‐day effects. Time of sacrifice and recording should therefore be considered a relevant experimental variable in astrocyte imaging and slice physiology studies and should systematically be reported, alongside other standard parameters such as age, sex, and brain regions.

Whether the sex‐ and ZT‐dependent differences observed here would be recapitulated in vivo remains an open question. As noted by (Khakh and McCarthy 2015), the core observations on Ca^2+^ signaling established in situ are largely preserved in vivo in awake mice, suggesting that ex vivo preparations remain informative of physiological astrocyte states. Nevertheless, in vivo recordings would be required to confirm our findings.

### 4.6 Considerations on the Ex Vivo Approach

The use of acute brain slices, while enabling high‐resolution Ca^2+^ imaging with precise experimental control, introduces inherent limitations. Slice preparation inevitably involves mechanical injury, loss of long‐range network connectivity, and disruption of the systemic milieu. Astrocytes are known to exhibit reactive changes rapidly following slicing, and it is therefore possible that the ZT‐dependent differences observed here partly reflect circadian variation in astrocytic responses to the slicing procedure itself, rather than, or in addition to, differences in intrinsic baseline astrocytic activity. In this regard, Mattei et al. recently demonstrated that microglial responses to pro‐inflammatory challenges vary with ZT in vivo, raising the analogous possibility that astrocyte reactivity to tissue injury may similarly depend on time of day (Mattei et al. 2024).

An additional technical consideration concerns recording temperature. Experiments were performed at 32°C, which is standard practice in slice physiology but remains below physiological brain temperature. Given that spontaneous astrocytic Ca^2+^ activity is known to be temperature‐sensitive (Schipke et al. 2008), we cannot rule out the possibility that the absolute values of the Ca^2+^ parameters reported here differ from those that would be observed at physiological temperature. Critically, however, both ZT groups were recorded under identical conditions, and we therefore do not expect this limitation to affect the relative ZT‐ and sex‐dependent differences that are the focus of the present study.

All Ca^2+^ events reported here also reflect spontaneous astrocytic activity, recorded in the absence of any pharmacological, electrical, or mechanical stimulation. In vivo, astrocytic Ca^2+^ activity is further shaped by ongoing neuronal activity and neuromodulatory inputs, including noradrenergic signaling from the locus coeruleus, discussed above in the context of ZT, and sex‐dependent priming, as well as by vigilance‐state‐dependent regulatory influences that are not fully preserved in acute‐slice preparations (Ding et al. 2013; Khakh and McCarthy 2015). The spontaneous activity recorded ex vivo, therefore, likely reflects intrinsic astrocytic properties and local circuit‐dependent dynamics rather than the full complement of in vivo modulatory influences. Nevertheless, spontaneous astrocytic Ca^2+^ activity and related gliotransmission have been implicated in the regulation of local synaptic transmission and plasticity (Pannasch and Rouach 2013; Sibille et al. 2015; Scharbarg et al. 2023), suggesting that the ZT‐ and sex‐dependent differences in spontaneous Ca^2+^ dynamics reported here may have functional consequences for local gliotransmission and VLPO circuit regulation.

We therefore acknowledge that the ex vivo signatures reported here, while robust and reproducible, may not map one‐to‐one onto in vivo VLPO astrocyte dynamics. Core features of astrocyte Ca^2+^ signaling recorded in acute slices, including spontaneous fluctuations and activity‐dependent Ca^2+^ elevations, are also detected in vivo in awake mice (Khakh and McCarthy 2015), suggesting that ex vivo preparations remain informative of physiological astrocyte states. Nevertheless, direct in vivo confirmation would ultimately be required, and the deep location and small size of the VLPO make such recordings particularly invasive and technically challenging.

In this context, our findings reveal that sex‐dependent astrocytic network dynamics within sleep–wake regulatory nuclei may contribute to sex‐specific sleep regulation. In women, the greater sleep needs phenotype could rely on the increased vulnerability to sleep disruption if sleep circuits require stronger or more tightly coordinated glial support to maintain sleep stability, particularly across hormonal transitions. These results could provide a mechanistically grounded direction for future translational research targeting glial physiology.

Overall, our findings indicate that VLPO astrocytes display distinct sex‐ and time‐of‐day‐dependent patterns of Ca^2+^ activity. Males primarily exhibit changes in event kinetics while maintaining a relatively stable functional network organization, whereas females show stronger recruitment of astrocytic activity together with increased synchronization and altered network organization at ZT‐14. Given established sex differences in sleep architecture and hormone‐dependent regulation in rodents, these findings reveal sex‐ and time‐of‐day‐dependent differences in VLPO astrocytic Ca^2+^ dynamics and functional network organization.

## Author Contributions

Conceptualization and supervision Armelle Rancillac. Data acquisition: Félix Camille Bellier. Formal Analysis: Lou Zonca. Figures: Armelle Rancillac. Writing, Review and Editing: Armelle Rancillac. Critically revised the manuscript, all authors; Funding Acquisition: Nathalie Rouach.

## Funding

This work was funded by CNRS, INSERM, Collège de France, and grants from ANR (AstroXcite) and the Major Research Program of PSL Research University “PSL‐Neuro” launched by PSL Research University and implemented by ANR (ANR‐10‐IDEX‐0001) to N.R., and from the Biosigne doctoral school (Paris Saclay University) to FCB.

## Conflicts of Interest

The authors declare no conflicts of interest.

## Supporting information


**Video S1:** Spontaneous astrocytic Ca^2+^ dynamics in the VLPO of a male mouse at ZT‐2. Representative two‐photon Ca^2+^ imaging recording of GCaMP6f‐expressing VLPO astrocytes from a male mouse sacrificed at ZT‐2, during the light phase (elevated sleep propensity).

**Video S2:** Spontaneous astrocytic Ca^2+^ dynamics in the VLPO of a male mouse at ZT‐14. Representative two‐photon Ca^2+^ imaging recording of GCaMP6f‐expressing VLPO astrocytes from a male mouse sacrificed at ZT‐14, during the dark phase (elevated wake propensity).

**Video S3:** Spontaneous astrocytic Ca^2+^ dynamics in the VLPO of a female mouse at ZT‐2. Representative two‐photon Ca^2+^ imaging recording of GCaMP6f‐expressing VLPO astrocytes from a female mouse sacrificed at ZT‐2, during the light phase (elevated sleep propensity).

**Video S4:** Spontaneous astrocytic Ca^2+^ dynamics in the VLPO of a female mouse at ZT‐14. Representative two‐photon Ca^2+^ imaging recording of GCaMP6f‐expressing VLPO astrocytes from a female mouse sacrificed at ZT‐14, during the dark phase (elevated wake propensity).

**Data S1:** Video legends.

## Data Availability

The data that support the findings of this study are available from the corresponding author upon reasonable request.
