# Supplementary material for: Female Mice Show Stronger Time‐of‐Day Modulation of Astrocytic Ca2+ Activity in the Sleep‐Regulatory Ventrolateral Preoptic Nucleus
Source: Glia. 2026 Jun 7;74(8):e70181. doi: 10.1002/glia.70181 (PMC13243727; doi:10.1002/glia.70181)
Supplement: Supplementary file 5 — Data S1: Video legends. [file GLIA-74-0-s003.docx]

• **Video S1** - Male, ZT-2

• **Video S2** - Male, ZT-14

• **Video S3** - Female, ZT-2

• **Video S4** - Female, ZT-14

**Video S1. Spontaneous astrocytic Ca²⁺ dynamics in the VLPO of a male mouse at ZT-2.** Representative two-photon Ca²⁺ imaging recording of GCaMP6f-expressing VLPO astrocytes from a male mouse sacrificed at ZT-2, during the light phase (elevated sleep propensity).

**Video S2. Spontaneous astrocytic Ca²⁺ dynamics in the VLPO of a male mouse at ZT-14.** Representative two-photon Ca²⁺ imaging recording of GCaMP6f-expressing VLPO astrocytes from a male mouse sacrificed at ZT-14, during the dark phase (elevated wake propensity).

**Video S3. Spontaneous astrocytic Ca²⁺ dynamics in the VLPO of a female mouse at ZT-2.** Representative two-photon Ca²⁺ imaging recording of GCaMP6f-expressing VLPO astrocytes from a female mouse sacrificed at ZT-2, during the light phase (elevated sleep propensity).

**Video S4. Spontaneous astrocytic Ca²⁺ dynamics in the VLPO of a female mouse at ZT-14.** Representative two-photon Ca²⁺ imaging recording of GCaMP6f-expressing VLPO astrocytes from a female mouse sacrificed at ZT-14, during the dark phase (elevated wake propensity).
